# Characteristics of language impairment in Parkinson’s disease and its influencing factors

**DOI:** 10.1186/2047-9158-4-2

**Published:** 2015-01-27

**Authors:** Lin Liu, Xiao-Guang Luo, Chui-Liang Dy, Yan Ren, Yu Feng, Hong-Mei Yu, Hong Shang, Zhi-Yi He

**Affiliations:** Neurology Department of First Affiliated Hospital of China Medical University, 155 Nanjing Bei Street, Heping District, Shenyang, 110001 China; Department of Laboratory Medicine of First Affiliated Hospital of China Medical University, 155 Nanjing Bei Street, Heping District, Shenyang, 110001 China

**Keywords:** Aphasia quotient, Language function deterioration rate, Language impairment, Parkinson’s disease, Western aphasia battery

## Abstract

**Background:**

Language impairment is relatively common in Parkinson’s disease (PD), but not all PD patients are susceptible to language problems. In this study, we identified among a sample of PD patients those pre-disposed to language impairment, describe their clinical profiles, and consider factors that may precipitate language disability in these patients.

**Methods:**

A cross-sectional cohort of 31 PD patients and 20 controls were administered the Chinese version of the Western Aphasia Battery (WAB) to assess language abilities, and the Montreal Cognitive Assessment (MoCA) to determine cognitive status. PD patients were then apportioned to a language-impaired PD (LI-PD) group or a PD group with no language impairment (NLI-PD). Performance on the WAB and MoCA was investigated for correlation with the aphasia quotient deterioration rate (AQDR).

**Results:**

The PD patients scored significantly lower on most of the WAB subtests than did the controls. The aphasia quotient, cortical quotient, and spontaneous speech and naming subtests of the WAB were significantly different between LI-PD and NLI-PD groups. The AQDR scores significantly and positively correlated with age at onset and motor function deterioration.

**Conclusion:**

A subset group was susceptible to language dysfunction, a major deficit in spontaneous speech. Once established, dysphasia progression is closely associated with age at onset and motor disability progression.

## Background

Parkinson’s disease (PD) is a neurodegenerative disorder characterized by progressive loss of dopaminergic neurons, primarily in the substantia nigra pars compacta [1]. Incidence rates of PD increase rapidly in people older than 60 years [2], and for those older than 55 years the cumulative risk is 4.9% [3]. PD patients most commonly present with motor impairments, including the ability to communicate verbally [4], but problems with cognition and other systems also occur [5, 6]. These can emerge at any stage of the disease and become worse as the disease develops, with significant adverse effects on quality of life.

Language problems afflict the majority of PD patients, especially in voice and articulation [7]. Spontaneous speech is severely reduced [8], making verbal communication slower and less accurate, and deficits in verb inflection [9, 10], verbal fluency [11], and verb generation [12] are common. The etiology of language deficits in PD is not definitive; most studies have focused on an association with handicapped cognition. However, while some researchers hypothesize that language abilities in PD correlate with cognitive abilities [13, 14], others more specifically connect language deficits to working memory and executive function [15]. In addition, clinical observations have suggested that some degree of depression can affect language performance. Starkstein et al. [16] found that patients with major depression performed significantly worse than non-depressed patients on all aspects of neuropsychological function tests, including language tasks. However, another study suggested no difference in language function among patients with minor depression or major depression and the control group [17]. Thus far, there is no consensus on how major depression affects language function.

Although language impairment in PD is common, not all patients are affected, even over the long-term progression of the disease [18]. The clinical profiles or features that might differentiate patients who are predisposed to language impairment have been generally overlooked, and factors that precipitate language disability remain elusive. In this study, we wanted to conclude with a clinical profile of PD patients predisposed to language impairment, and explore factors related to rapid progression of language impairment in PD. Therefore, we evaluated the clinical profiles of PD patients with language impairment relative to those without impairment, and of healthy controls, noting differences in demographics, motor abilities, and cognitive states. We used the Western Aphasia Battery (WAB) [19], a tool routinely used in many neurological diseases, to evaluate adult language function [20, 21].

## Methods

### Patients

The Ethics Review Board of First Affiliated Hospital of China Medical University (Shenyang, China) approved the protocol of the study. Thirty-one PD patients (18 men and 13 women, aged 63.35 ± 8.84 y) at the Parkinson’s Disease Outpatient Center of the hospital were enrolled between May 2013 and October 2013. None of the patients had a family history of PD, and all met the criteria for idiopathic PD, according to the UK Parkinson’s Disease Society Brain Bank clinical diagnostic criteria [22]. All patients were given the best medications for PD. Patients were excluded if they had clinical features of Parkinson’s-plus syndrome. None of the patients or their caregivers complained of obvious cognitive decline in the patients, and all patients were considered non-demented.

Twenty healthy individuals (age 64.85 ± 8.27 y), most of whom were spouses of the PD patients and were comparable for age, gender, and educational level, were selected as a control group. None of the controls expressed cognitive complaints or reported a history of neurological or psychiatric illnesses, and none had a history of severe cardiovascular, renal, or hepatic disorders. All PD patients and healthy controls were right-handed.

The 17-item Hamilton Depression Rating Scale (HAMD-17) [23] was administered to all prospective participants to identify and measure the severity of depression symptoms, and individuals with scores >15 were excluded from the current study.

Demographic data were obtained for each participant, as well as clinical information consisting of Unified Parkinson’s Disease Rating Scale (UPDRS) scores, Hoehn Yahr stage [24] for motor function, and Montreal Cognitive Assessment (MoCA) [25] scores for cognitive function.

### Outcome measures

Defining the type and degree of language impairment in PD patients is hampered by a lack of generally recognized clinical criteria. The WAB [19] is an accepted method for assessing language abilities in many neurological diseases including stroke [26, 27], amyotrophic lateral sclerosis [28], corticobasal degeneration syndrome [29], and brain tumor [30]. The WAB aphasia quotient (AQ) provides a global measure of aphasia severity [31] and was found appropriate for assessing non-fluent aphasia [32]. Therefore, we employed the WAB to assess the language abilities of our subjects.

The WAB includes both linguistic and nonlinguistic subtests. The former comprises subtests for spontaneous speech, auditory comprehension, repetition, and naming. The nonlinguistic portion includes subtests for reading, writing, praxis, and construction. Based on the results and the formula (below), the AQ, performance quotient, and cortical quotient were determined: the AQ reflects the severity and type of aphasia; the performance quotient represents the nonlinguistic function of the brain; and the cortical quotient gives an overall picture of cognitive status [33].

The AQ (range 0–100) is derived from the linguistic subtests as follows: AQ = (spontaneous speech score + auditory comprehension score/20 + repetition score/10 + naming score/10) × 2. Since there is no standard AQ cutoff value for PD patients, we used the scores of the control group to establish a cutoff value. Patients were considered language impaired if they scored at least two standard deviations below the mean AQ value for the age- and education- comparable controls. A similar approach to setting cutoff points has been used in previous studies to define impairment in neuropsychological tests [26, 27]. Based on the results from the controls, a value of 94.5 was defined as the cutoff AQ value. AQ values ≥ 94.5 were considered normal; individuals with scores < 94.5 were defined as exhibiting language impairment.

To evaluate the rate of decline in language ability of PD patients, the following formula for the AQ deterioration rate (AQDR) was applied: AQDR = (94.5 – AQ)/(disease duration in years). Patients scoring > 94.5 were assigned a value of exactly 94.5 to avoid negative values. A similar approach was used previously [26, 27, 34].

The performance quotient, which combined results of the reading, writing, praxis, and construction subtests of the WAB, was used as a reflection of the non-spoken language function of patients. The cortical quotient was calculated to represent the general cognitive function, according to the formula: cortical quotient = (AQ/2) + performance quotient + (auditory comprehension score/20).

For PD patients, motor performance was evaluated using Parts II and III of the UPDRS, and cognition was evaluated by the Beijing version of the MoCA (the specific test forms and instructions for Beijing version are available at the MoCA official web-site http://www.mocatest.org/). The following formula was used to assess the motor function deterioration rate in points per year: Motor function deterioration rate, points/y = (UPDRS III, points)/(disease duration, y). To reduce the variability in motor function between optimally treated patients and patients who may have been over- or under-medicated, the UPDRS rating of each patient was determined at a time when the patient had been without PD medications for at least 12 hours.

### Statistical analyses

Statistical analyses were performed using the software program SPSS 17.0 (SPSS, Chicago, IL). Two independent sample *t*-tests for normally distributed variables (including age, age at onset, HAMD-17 scores, and MoCA total score), or the Mann–Whitney U test for non-normally distributed variables, were used to assess the differences between the PD and control groups, and between the language-impaired PD (LI-PD) and no language impairment PD (NLI-PD) groups. Linguistic features of the LI-PD, NLI-PD, and control groups were analyzed using analysis of variance by setting the MoCA score as a covariant.

Univariate and multivariate regression analyses were performed to determine the association between AQDR and the demographic and motor features of PD patients, and between the AQ and cognitive status as evaluated by MoCA. Results are reported as mean ± standard deviation. Values of *P* < 0.05 were considered statistically significant.

## Results

### Language abilities of PD patients were impaired compared with controls

The language abilities of the 31 PD patients and 20 controls were assessed using the WAB (Table 1). The mean AQ score of the PD group (93.11 ± 4.55) was significantly lower than that of the control group (98.07 ± 1.75; *P* < 0.001). In addition, the PD patients scored significantly lower on some of the subtests of the WAB (*P* < 0.05), namely those for spontaneous speech, repetition, writing, praxis and construction, performance quotient, and cortical quotient.

**Table 1:** **Difference of WAB subtests in PD patients and controls** ^**a**^

		PD patients ^b^	Controls ^c^	***P***-value
Gender	Men	18	12	0.891
	Women	13	8	
Age, y		63.35 ± 8.84 (44–77)	64.85 ± 8.27 (51–76)	0.543
Formal education, y		10.74 ± 3.08	10.95 ± 2.69	0.800
MoCA total scores		21.00 ± 4.341	25.80 ± 1.642	0.000^d^
HAMD-17 score		8.42 ± 2.29	7.45 ± 2.44	0.157
Subtests of WAB	Spontaneous speech	18.19 ± 1.92	19.75 ± 0.55	0.000^d^
	Auditory comprehension	191.10 ± 8.45	194.50 ± 2.80	0.656
	Repetition	92.35 ± 4.71	97.70 ± 3.37	0.000^d^
	Naming	95.97 ± 4.62	98.30 ± 2.27	0.078
	Reading	91.14 ± 9.20	95.85 ± 7.05	0.061
	Writing	74.55 ± 13.92	89.80 ± 5.98	0.000^d^
	Praxis and construction	80.81 ± 9.76	87.80 ± 7.45	0.013^e^
	Aphasia quotient	93.11 ± 4.55 (81.9-99.3)	98.07 ± 1.75 (95.9-100.0)	0.000^d^
	Score < 94.5, n (%)	17 (54.8%)	0 (0%)	NA
	Performance quotient	34.67 ± 2.65	37.26 ± 1.65	0.001^d^
	Cortical quotient	91.03 ± 4.68	96.07 ± 2.47	0.000^d^

^a^Data are expressed as numbers or as mean ± standard deviation, with percentages or ranges in parentheses; ^b^n = 31; ^c^n = 20; ^d^ *P* < 0.01; ^e^ *P* < 0.05.

Levene’s test for equality of variances: MoCA total score F = 8.14, P = 0.007; Spontaneous speech F = 13.76, P = 0.001; Repetition F = 2.52 P = 0.119; Writing F = 13.52, P = 0.001; Praxis and construction F = 2.40 P = 0.129; Aphasia quotient F = 13.65 P = 0.001; Performance quotient F = 2.59 P = 0.106; Cortical quotient F = 6.57 P = 0.014.

### Demographic features and MoCA performance in PD patients with and without language impairment

Seventeen of the PD patients (54.8%) scored ≥2 standard deviations below the control mean AQ value (cutoff value = 94.5) and therefore comprised the LI-PD group (Table 2). The remaining PD patients (n = 14; 45.2%) had AQ scores > 94.5 and were considered the NLI-PD group.

**Table 2:** **Demographic features in LI-PD, NLI-PD and control groups** ^**a**^

	LI-PD group (n = 17)	NLI-PD group (n = 14)	Control group (n = 20)	***P***, LI-PD cf. NLI-PD	***P***, LI-PD cf. control	***P***, NLI-PD cf. control
Men	12	6	12	0.157	0.731	0.487
Women	5	8	8			
Age, y	63.59 ± 8.62	63.07 ± 9.43	64.85 ± 8.27	0.843	0.653	0.564
Age at onset, y	55.06 ± 10.92	56.36 ± 10.95	NA	0.781	NA	NA
Formal education, y	10.53 ± 2.67	11.00 ± 3.60	10.95 ± 2.69	0.780	0.637	0.983
Disease duration, y	8.53 ± 4.49	6.71 ± 3.29	NA	0.327	NA	NA
Hoehn-Yahr stage	2.50 ± 0.55	2.57 ± 0.39	NA	0.415	NA	NA
UPDRS part II	15.69 ± 8.39	11.79 ± 4.87	NA	0.138	NA	NA
UPDRS part III	38.87 ± 12.11	32.86 ± 8.76	NA	0.135	NA	NA
HAMD-17 scores	8.88 ± 2.34	7.86 ± 2.18	7.45 ± 2.44	0.691	0.208	1.000
MoCA scores	19.60 ± 4.75	22.75 ± 3.14	25.80 ± 1.64	0.142	0.000^b^	0.021^b^

^a^Data are expressed as numbers or mean ± standard deviation; NA = Not applicable; LI-PD = language impairment Parkinson’s disease; NLI-PD = no language impairment Parkinson’s disease; cf. = confer (compare); ^b^ *P* < 0.05.

F value of MoCA scores between groups is 15.32, and P value is 0.000.

No significant difference for any demographic variable was found between the LI-PD and NLI-PD patients, including age, age at onset, level of education, disease duration, Hoehn-Yahr stage, UPDRS part II and part III scores, and HAMD-17 scores. This suggests that the influence of demographics on the incidence of language impairment in PD was minimal.

We also assessed differences in the characteristics of language function between the LI-PD and NL-PD groups as evaluated by the WAB (Table 3). We set the MoCA score as a covariant to exclude the influence of cognitive state. The result showed that between the LI-PD and NLI-PD groups the differences were significant with regard to AQ, cortical quotient of the WAB, and the scores of the spontaneous speech and naming subtests of the WAB. No significance was found between the NLI-PD group and the controls. These results suggest that the language abilities and clinical profiles of PD patients without language impairment are basically the same as those of the controls, and the language impairments measured for PD patients as a single group are mainly due to the subset of PD patients prone to having language deficits. Thus language deficits are unique to this subset of PD patients.

**Table 3:** **Characteristics of language function in LI-PD, NLI-PD and control groups** ^**a**^

Subtests of WAB	LI-PD ^b^	NLI-PD ^c^	Control ^d^	F(df), LI-PD cf. NLI-PD	***P***, LI-PD cf. NLI-PD	F(df), LI-PD cf. control	***P***, LI-PD cf. control	F(df), NLI-PD cf. control	***P***, NLI-PD cf. control
Spontaneous speech	17.06 ± 1.92	19.57 ± 0.51	19.75 ± 0.55	24.343 (1, 28)	0.000^e^	28.736 (1, 34)	0.000^e^	0.250 (1, 31)	0.621
Auditory comprehension	189.24 ± 9.26	195.14 ± 5.83	194.50 ± 2.80	2.271 (1, 28)	0.143	0.965 (1, 34)	0.333	0.377 (1, 31)	0.544
Repetition	92.00 ± 4.96	92.79 ± 4.51	97.70 ± 3.37	0.218 (1, 28)	0.644	3.636 (1, 34)	0.065	3.657 (1, 31)	0.065
Naming	94.06 ± 5.06	98.29 ± 2.70	98.3 ± 2.27	4.258 (1, 28)	0.048^e^	5.238 (1, 34)	0.032^e^	0.606 (1, 31)	0.442
Reading	89.20 ± 9.25	92.75 ± 9.24	95.8 ± 7.05	0.556 (1, 28)	0.465	1.102 (1, 34)	0.303	0.017 (1, 31)	0.897
Writing	69.75 ± 10.86	78.51 ± 15.33	89.80 ± 5.98	1.877 (1, 28)	0.187	13.410 (1, 34)	0.001^e^	1.824 (1, 31)	0.187
Praxis and construction	78.44 ± 9.28	82.58 ± 10.13	87.8 ± 7.45	0.506 (1, 28)	0.486	0.392 (1, 34)	0.537	0.013 (1, 31)	0.908
Aphasia quotient	89.97 ± 3.69	96.94 ± 1.47	98.07 ± 1.75	34.835 (1, 28)	0.000^e^	36.259 (1, 34)	0.000^e^	0.434 (1, 31)	0.515
Performance quotient	33.83 ± 2.06	35.30 ± 2.94	37.26 ± 1.65	1.142 (1, 28)	0.299	5.294 (1, 34)	0.030^e^	0.407 (1, 31)	0.528
Cortical quotient	87.88 ± 4.20	93.40 ± 3.57	96.07 ± 2.47	9.945 (1, 28)	0.005^e^	14.206 (1, 34)	0.001^e^	0.483 (1, 31)	0.493

^a^Data are expressed as numbers or mean ± standard deviation; ^b^n = 17; ^c^n = 14; ^d^n = 20; ^e^ *P*< 0.05.

LI-PD = langue impairment Parkinson disease; NLI-PD = no language impairment Parkinson’s disease; cf. = confer (compare); df = degree of freedom.

### Language deficit is a feature independent of the cognitive state in LI-PD patients

The MoCA is a sensitive method for measuring cognitive function in PD patients, with good intra-class correlation and inter-rater reliability [25, 35]. To clarify whether language disorder is associated with cognitive impairment, we compared the MoCA scores of all the groups, and also analyzed the degree of correlation in language impairment PD patients between AQ and MoCA scores or its subtests (Table 4).

**Table 4:** **Correlation of AQ and MoCA scores for LI-PD patients**

MoCA score	***r***	***P***-value
Total	0.027	0.923
Lines	−0.077	0.785
Copy cube	0.098	0.728
Clock drawing test	−0.012	0.967
Naming	−0.164	0.560
Digit span forward	0.023	0.938
Digit span backward	0.140	0.620
Alertness	−0.174	0.534
Calculation	−0.258	0.354
Language repeat	0.392	0.148
Verbal fluency	0.314	0.254
Abstraction	−0.139	0.622
Delayed recall	0.042	0.881
Orientation	−0.289	0.296

LI-PD = language impairment Parkinson’s disease.

Our results showed that the MoCA scores of both PD groups were significantly lower compared with the controls (Table 2), while the differences in cognitive ability were not significantly different between the LI-PD and NLI-PD groups. This implies that even though language is a component of cognition, PD-related language impairment is a specifically isolated feature independent of overall cognitive status. The conclusion was further supported by the finding that among LI-PD patients, there was no significant correlation between the total MoCA score (or each of the MoCA subsets) and AQ (Table 4).

We also investigated whether a correlation exists between depression and language function in PD patients, and found no significant correlation (data not shown).

### Later age at onset is an independent risk factor for faster language dysfunction in LI- PD patients

To determine risk factors that influence the rate of language decline in PD patients, we used univariate or multivariate regression model analysis of the available clinical features of the 17 LI-PD patients (i.e., AQ ≤ 94.5). The parameters consisted of age at onset, education, disease duration, Hoehn-Yahr stage, and MoCA total score. Univariate linear regression analysis showed that the AQDR correlated significantly with both age at onset (B = 0.036, *P* = 0.037) and the deterioration rate of motor function (B = 0.167, *P* = 0.006). However, subsequent multivariate regression analysis revealed that only the age at onset was an independent factor for faster deterioration of language ability (B = 0.036, *P* = 0.037). Thus, the older the patient at PD onset, the faster language dysfunction progressed (Table 5).

**Table 5:** **Correlation of AQDR with demographic features of LI-PD patients**

	Univariate analysis			Multivariate analysis		
	B (95% CI)	F (df)	***P***-value	B (95% CI)	F (df)	***P***-value
Age at onset, years	0.036 (0.002, 0.069)	5.463 (1, 15)	0.037*	0.036 (0.002-0.069)	5.463 (1, 15)	0.037*
Motor function deterioration rate	0.167 (0.056, 0.278)	10.973 (1, 15)	0.006*			
Education, years	−0.015 (−0.173, 0.144)	0.037 (1, 15)	0.846			
Hoehn-Yahr stage	0.188 (−0.620, 0.997)	0.259 (1, 15)	0.625			
MoCA total score	0.012 (−0.090, 0.115)	0.069 (1, 15)	0.797			

LI-PD = language impairment Parkinson’s disease; CI = confidence interval; df = degree of freedom; *P < 0.05.

## Discussion

Recognizing that not all PD patients suffer from language disabilities, in the present study we attempted to characterize the subset of PD patients who do suffer verbal communication problems and determine factors that influence their susceptibility and rate of language deterioration. We found that the language abilities of the PD patients, as a group, had suffered relative to those of the healthy controls comparable for education and age. However, further investigation showed that findings regarding language impairment were based predominantly on a subset of PD patients considered LI-PD by the WAB scores, while the linguistic profiles of the remaining NLI-PD patients and controls were virtually identical. Furthermore, the language disorder of the LI-PD patients was independent of their cognitive status, suggesting a unique mechanism underlying language disorder in PD. We also determined that later age at onset was a risk factor for faster language deterioration.

The spontaneous speech subtest of the WAB accounts for 40% of the WAB assessment, and is an evaluation of information content and fluency [36]. A maximum score requires normal fluency and complete content, without hesitations or difficulties finding words, despite the anxiety of being tested. The poor performance of PD patients on the spontaneous speech portion of the WAB reflects their difficulty expressing themselves verbally. In the present study the spontaneous speech measure was the most important for discriminating the LI-PD and NLI-PD patients, as it accounted for 70% of the difference between the two groups; the scores between the NLI-PD patients (19.57 ± 0.51) and controls (19.75 ± 0.55) were virtually identical (*P* = 1.000). This suggests that as the most discriminating indicator of language disability, the spontaneous speech score is also the most sensitive for revealing language impairment in the subset of PD patients susceptible to language dysfunction.

In our study, the total MoCA score for all the PD patients together was significantly lower than that of the controls. This is consistent with the commonly recognized prevalence of cognitive impairment in PD [37]. Many studies have shown that impairments in cognitive ability could influence language function [13, 14, 38]. Based on this reasoning, we analyzed the correlation between cognition and language function. However, the MoCA scores of the LI- PD and NLI-PD patients were similar, and for the LI-PD there was no correlation between the AQ and MoCA scores (or between the AQ and any of the MoCA subtests). Therefore, even though language is a component of cognition, language dysfunction in PD is not associated with cognitive status, but rather is a relatively unique feature of a subset of PD patients predisposed to language problems.

This suggests that cognition is not the primary factor influencing language impairment, but that other factors are involved, or language impairment is simply due to the nature of the disease. Altmann and Troche [15] also concluded that there may be neural circuits, primarily used in language production, that are independently damaged in PD.

The pathophysiology that leads to language disorders may develop as Parkinson’s disease progresses, but for non-susceptible PD patients language ability may be kept relatively intact. Interestingly, the mean MoCA score of the healthy controls was only 25.8 ± 1.64, with 55% scoring less than 26. This is in accord with our previous study [34]. Through interviews with the families, we confirmed the healthy cognitive state of all our 20 controls. We thus conclude that 26 should not be a cutoff value in our group and attribute this difference to the different cultural background of these patients. In some studies, even lower MoCA scores for the controls were reported [39].

To identify risk factors for PD patients with language impairment, we compared the demographic features of LI-PD and NLI-PD patients. We found no significant differences between these groups with regard to age, age at onset, level of education, disease duration, or disease severity. This is similar to the results of Miller et al. [40].

While we contend that there is a discrete subset of PD patients susceptible to language deficits, even within the LI-PD group the rate progression of language disability varied widely, making risk factors difficult to determine. We thus continued to investigate the factors that influenced the rate of deterioration in the LI-PD patients, as reflected by the AQDR. The univariate linear regression analysis showed that AQDR correlated significantly with both age at onset (B = 0.036, *P* = 0.037) and the deterioration rate of motor function (B = 0.167, *P* = 0.006). However, according to the multivariate regression analysis, only age at onset was a significant risk factor. Therefore, for the language-impaired PD patient, age at onset of PD is an independent risk factor for the deterioration rate in language ability; that is, the later the age at onset of PD, the faster the rate of language dysfunction. This is in accord with a study by Locascio et al. [41], in which language deterioration in PD was related to late onset of the disease only. Yet, our study went a step further and isolated late onset as an independent risk factor.

In general, later-onset PD patients have faster motor deterioration, more severe motor symptoms, and rapid cognitive impairment [34, 42–46]. Some researchers have speculated that, unlike older patients, those younger have functional reserves that can compensate for impairments in certain other functions [47], which explains the effects associated with late onset of PD. Functional neuroimaging studies in healthy young and older participants indicate that older people have more bilateral activation [48]. Two theories are cited to explain this finding: dedifferentiation of activation or compensatory activation in older people. The dedifferentiation theory posits that older persons have difficulty recruiting specialized neural mechanisms. The compensatory hypothesis asserts that older persons employ homologous lateral structures to bolster impaired processing.

The design of the present pilot study imposed some limitations on its findings. Firstly, the MoCA provides only a rough estimate of cognitive function compared with other cognitive scales, and may not provide a full and accurate assessment of each of the cognitive domains. Secondly, we used the equation AQDR = (94.5 – AQ)/(disease duration in years) to calculate the AQ deterioration rate in patients with PD. Indeed, AQDR is unable to truly reflect the pattern of deterioration in patients’ language function, but only the average rate of deterioration. Long-term follow-up of patients is essential for the study of the pattern of language function deterioration. Thirdly, as an explorative study that relied on small and convenient samples, the subjects may not be representative of the general PD population, and larger samples are required to confirm or modify our results.

Our work constitutes a preliminary characterization and assessment of factors influencing language impairment in PD. Yet, our results strongly indicate that language disability in PD applies only to a distinct subset of PD patients, and late onset is an independent risk factor. Identifying the PD patients who are at high risk of language dysfunction and establishing the factors that influence language disorder, is important for improving the quality of life of these patients.

## Consent

Written informed consent was obtained from the patient for the publication of this report and any accompanying images.
